# Comprehensive Analysis of the Immune Infiltrates and Aberrant Pathways Activation in Atherosclerotic Plaque

**DOI:** 10.3389/fcvm.2020.602345

**Published:** 2021-02-05

**Authors:** Hukui Han, Rongsheng Du, Panke Cheng, Jiancheng Zhang, Yang Chen, Gang Li

**Affiliations:** ^1^Department of Cardiology, Sichuan Provincial People's Hospital, University of Electronic Science and Technology of China, Chengdu, China; ^2^Department of Cardiology, West China Hospital, Sichuan University, Chengdu, China; ^3^Emergency Department, Sichuan Provincial People's Hospital, University of Electronic Science and Technology of China, Chengdu, China

**Keywords:** immune infiltrates, atherosclerotic plaque, coronary artery disease, pathways, immune microenvironment

## Abstract

Atherosclerosis is the pathological basis of many cardiovascular and cerebrovascular diseases. The development of gene chip and high-throughput sequencing technologies revealed that the immune microenvironment of coronary artery disease (CAD) in high-risk populations played an important role in the formation and development of atherosclerotic plaques. Three gene expression datasets related to CAD were assessed using high-throughput profiling. CIBERSORT analysis revealed significant differences in five types of immune cells: activated dendritic cells (DCs), T follicular helper cells (Tfhs), resting CD4+ T cells, regulatory T cells (Tregs), and γδ T cells. Immune transcriptome analysis indicated higher levels of inflammatory markers (cytolytic activity, antigen presentation, chemokines, and cytokines) in the cases than in the controls. The level of activated DCs and the lipid clearance signaling score were negatively correlated. We observed a positive correlation between the fraction of Tfhs and lipid biosynthesis. Resting CD4+ T cells and the activity of pathways related to ossification in bone remodeling and glutathione synthesis showed a negative correlation. Gamma delta T cells negatively correlated with IL-23 signaling activity. GSEA revealed a close association with the inflammatory immune microenvironment. The present study revealed that CAD patients may have an inflammatory immune microenvironment and provides a timely update on anti-inflammatory therapies under current investigation.

## Introduction

Coronary artery disease (CAD) leads to myocardial infarction (MI), ischemic cardiomyopathy, and arrhythmia, and it is the main source of cardiovascular morbidity, mortality, and the economic health burden worldwide (1). The past decade of research has provided a deeper understanding of the pathogenesis and treatment of CAD (2). Despite this success, little progress has been made on elucidating the function of the immune microenvironment and its therapeutic Implications (3). Growing evidence suggests that that many aforementioned risk factors, such as smoking habits, obesity, hypertension, insulin resistance, diabetes, stress, and hyperlipidemia are primarily responsible for CAD (4). A significant link exists between the pathophysiology of CAD and inflammatory mediators, immune cells, oxidative stress, lipid infiltration, extracellular matrix, and hormone metabolism (4–8).

High-throughput sequencing technologies examine the role of the immune microenvironment of CAD in high-risk populations in the formation and development of atherosclerotic plaques (APs) (4, 9–12). A growing number of studies indicate that cardiovascular events are determined by multiple biological processes that require different tailored therapeutic approaches, and future therapies for CAD should tackle the residual inflammatory process that statin therapy only partially addresses (13). Inhibitors of specific components of the immune microenvironment in atherosclerosis were developed for considerable treatment (13–15). For example, trials involving anti-cytokine therapy for atherosclerotic CAD targeted interleukin (IL)-1β, which plays numerous roles in atherogenesis, plaque growth, and subsequent rupture (16). Pre-clinical studies also revealed that the inhibition of IL-6 or its receptor achieved atheroprotective effects (17, 18).

Converging lines of evidence support the importance of the immune microenvironment in the initiation, progression, and vulnerability of atherosclerotic plaques. Therefore, we analyzed the immune microenvironment based on three CAD datasets. Several immune infiltration analyses were used to reveal the molecular mechanisms of various factors, such as immune cells, cytokines, chemokines, and abnormally activated pathways, in the occurrence and development of CAD and provide a theoretical basis for anti-atherosclerotic treatment strategies and the prevention of this disease.

## Materials and Methods

### Data Processing

**Figure 1:**
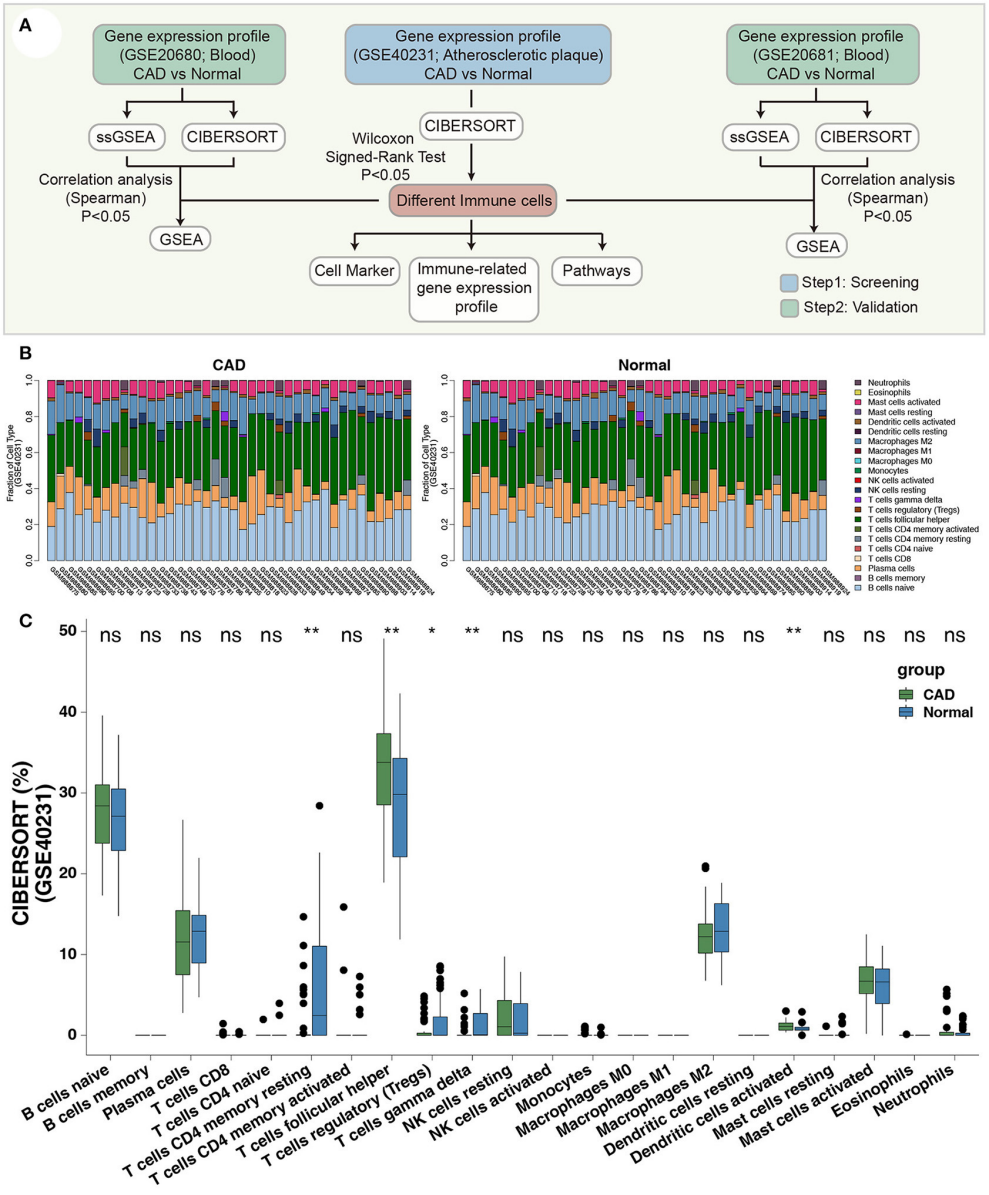
**(A)** The workflow of the present study. **(B)** The bar plot indicates the proportions of the 22 immune cells of each sample in GSE40231. **(C)** The proportions of immune cells of the CAD cases vs. the healthy controls in GSE40231 using CIBERSORT analysis. Wilcoxon signed-rank test (paired): **P < 0.01; *P < 0.05. CAD, coronary artery disease.

We collected three datasets related to CAD from the Gene Expression Omnibus database (GEO, https://www.ncbi.nlm.nih.gov/geo/) (19), for analysis using the GEOquery R package (20). The GSE40231 dataset based on the GPL570 platform included samples from 40 CAD patients (40 atherosclerotic aortic walls vs. internal mammary arteries) and was used to examine differences in immune filtration in APs. The GSE20681 dataset, based on GPL4133, included 99 whole blood samples from CAD patients and 99 whole blood samples from healthy controls. The GSE20681 samples were paired based on case/control status, age, and gender. GSE20680 included whole blood samples from 143 CAD patients and 52 healthy individuals and were both based on GPL4133. We used normalizeBetweenArrays of the limma R package to the normalize expression data in GSE40231, GSE20681, and GSE20680 (21). Figure 1A shows the workflow of our study.

### Immune Profiling Based on the CIBERSORT Algorithm

The CIBERSORT algorithm uses the LM22 gene sets to estimate the proportion of 22 infiltrating immune cells in samples based on the expression data (22). We used this algorithm based on the default parameters to calculate the relative proportion of 22 infiltrating immune cells in our samples.

We also used this LM22 gene set to compare differences in the detailed gene expression of the significantly altered immune cells. The genes related to cytolytic activity (CYT), antigen presentation, chemokines, and cytokines in CAD identified in previous studies were used to evaluate differences in immune function between the CAD and control groups (23, 24).

### Evaluation of the Abnormal Signaling Signature Based on Single-Sample Gene Set Enrichment Analysis (ssGSEA) and Gene Set Enrichment Analysis (GSEA)

The abnormal signaling score was calculated from expression data (GSE40231, GSE20681, and GSE20680) using the ssGSEA algorithm and the GSVA R package (25–27). Gene sets of pathways were collected from the MsigDB database (28). The limma R package was used to calculate the difference in gene expression (logFC) between the CAD and control groups in this study (21). GSEA was performed using the “clusterProfiler” R package and the Molecular Signatures Database (MSigDB) to annotate the dataset, and Gene Ontology (GO), Kyoto Encyclopedia of Genes and Genomes (KEGG) and Reactome terms were considered significant at *P* < 0.05 (29).

### Statistical Analysis

The Wilcoxon signed-rank test (paired) was used to compare differences in the relative proportion of infiltrating immune cells and differences in the immune-related gene expression profile (GEP) between the CAD and control groups. *P* < 0.05 was considered statistically significant, and all statistical tests were two-sided. R software (version 3.6) was used for statistical analyses. The R package ComplexHeatmap was used to visualize the heatmap (30). The R package ggplot2 was used to create the violin plot (31). The corrplot R package was used to visualize the correlation heatmap (32).

## Results

### Immune Cells in CAD Cases

To identify a robust immune infiltrating pattern, we normalized three gene expression datasets (GSE40231, GSE20681, and GSE20680) based on the normalizeBetweenArrays method of the limma R package (Supplementary Figure 1). The CIBERSORT algorithm was used to calculate the proportion of 22 immune cells based on the expression data of the discovery dataset (GSE40231; Figure 1B). Notably, the fractions of activated DCs and Tfhs were significantly higher in the CAD than in the control groups (Figure 1C). In contrast, the CAD group had fewer Tregs, γδ T cells, and resting CD4+ T cells (Figure 1C) than the control group. These results suggest that CAD exhibits inflammatory microenvironment patterns.

### Different GEPs Related to Immune Cells, CYT, Antigen Presentation, Chemokines, and Cytokines

**Figure 2:**
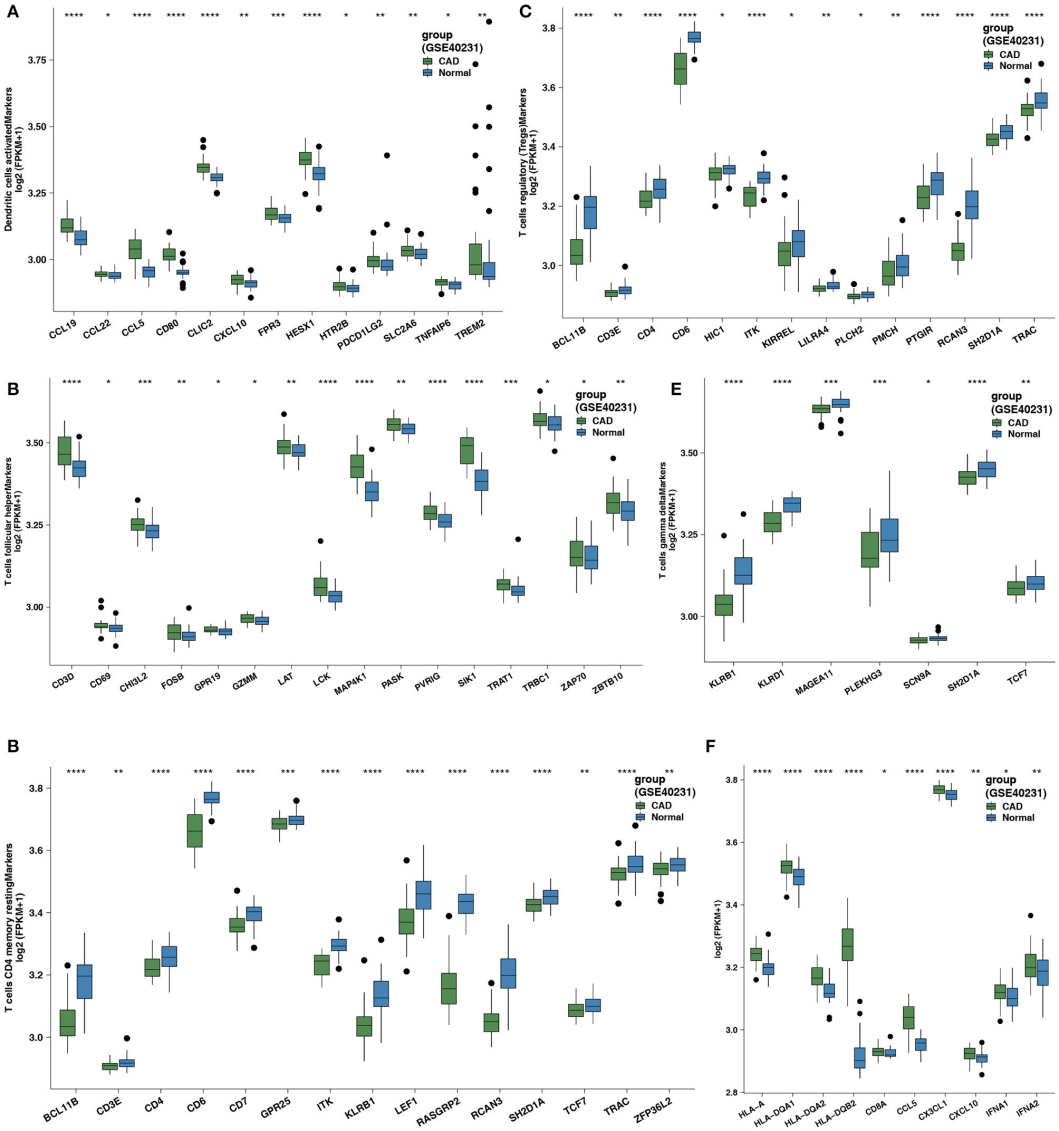
**(A)** Comparison of the expression of cell markers of activated DCs between the CAD cases vs. the healthy controls in GSE40231. **(B)** Comparison of the expression of cell markers of Tfhs between the CAD cases vs. the healthy controls in GSE40231. **(C)** Comparison of the expression of cell markers of Tregs between the CAD cases vs. the healthy controls in GSE40231. **(D)** Comparison of the expression of cell markers of resting CD4+ T cells between the CAD cases vs. the healthy controls in GSE40231. **(E)** Comparison of the expression of cell markers of γδ T cells between the CAD cases vs. the healthy controls in GSE40231. **(F)** Comparison of the expression of CYT-related, antigen-presentation, and proinflammatory-related genes (chemokine and cytokine) between the CAD cases vs. the healthy controls in GSE40231. DCs, dendritic cells; CAD, coronary artery disease; CYT, cytochrome; Tfhs, T follicular helper cells; Tregs, regulatory T cells; γδ T cells, gamma delta T cells. Wilcoxon signed-rank test: ****P < 0.0001; ***P < 0.001; **P < 0.01; *P < 0.05.

We focused on the comparison of five differential immune cell markers between the CAD and control groups. Two cell types in CAD (activated DCs and Tfhs) exhibited increased expression levels of immune cell-related genes (Figures 2A,B; all Wilcoxon signed-rank Ps < 0.05). Tregs, γδ T cells, and resting CD4+ T cells in the control group had low expression levels of immune cell-related genes (Figures 2C–E; all Wilcoxon signed-rank Ps < 0.05). We performed the Wilcoxon signed-rank test on the expression levels of genes related to immune function between the CAD and control groups and found that the GEPs related to CYT (CD8A), antigen presentation (HLA-A, HLA-DQA1, HLA-DQA2, and HLA-DQB1), chemokines (CCL5, CX3CL1, and CXCL10), and cytokines (IFNA1 and IFNA2) were significantly stronger in the CAD group than in the control group (all Wilcoxon signed-rank Ps < 0.05).

### Identification of Abnormal Pathways Related to Five Immune Cell Types in CAD

**Figure 3:**
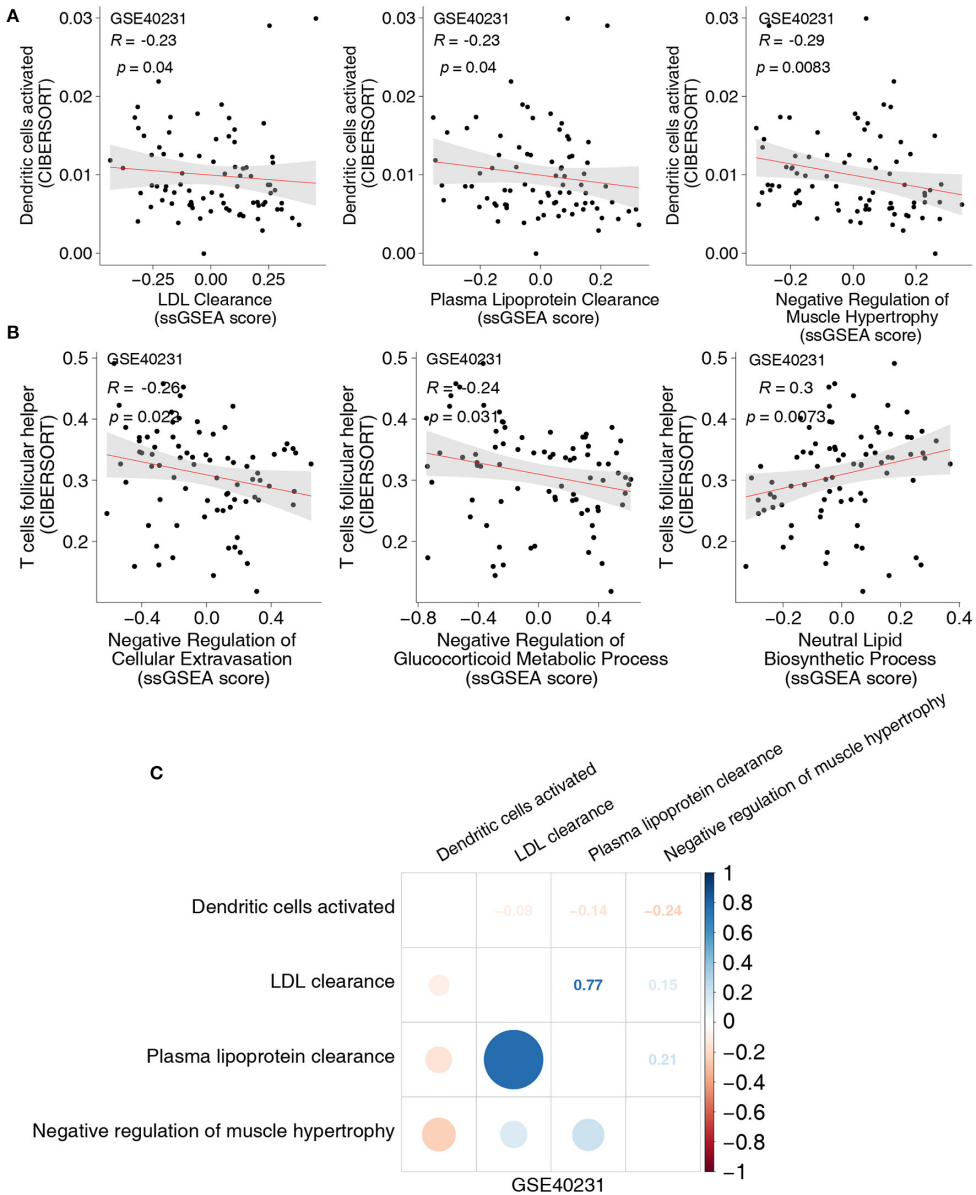
**(A)** The correlation between the proportions of activated DCs and the score of ssGSEA in GSE40231. **(B)** The correlation between the proportions of Tfhs and the score of ssGSEA in GSE40231. **(C)** The correlation heatmap of the proportions of activated DCs and the score of ssGSEA in each three-gene expression dataset (GSE40231). DCs, dendritic cells; Tfhs, T follicular helper cells; ssGSEA, enrichment analysis.

**Figure 4:**
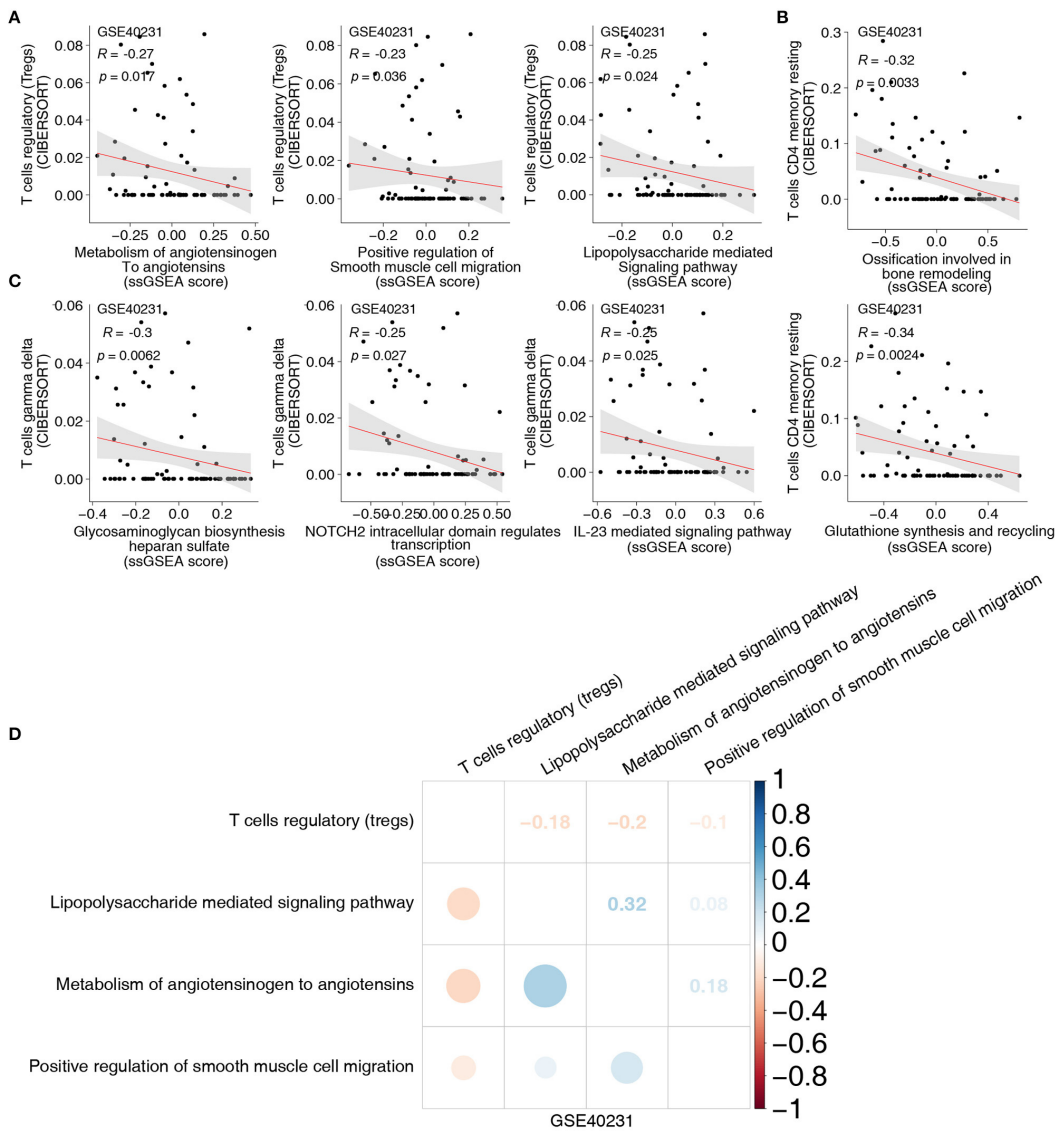
**(A)** The correlation between the proportions of Tregs and the score of ssGSEA. **(B)** The correlation between the proportions of resting CD4+ T cells and the score of ssGSEA. **(C)** The correlation between the proportions of γδ T cells and the score of ssGSEA. **(D)** The correlation heatmap of the proportions of Tregs and the score of ssGSEA in each three-gene expression dataset (GSE40231). Tregs, regulatory T cells; ssGSEA, enrichment analysis; γδ T cells, gamma delta T cells.

To uncover the different signaling signatures between the CAD and control groups, we performed ssGSEA to calculate the scores of the pathways of each sample using the GSVA R package. Spearman's correlation analysis was performed to determine the relationship between the score of the ssGSEA and the fraction of each of the five immune cells. The proportion of activated DCs and the activity of lipid clearance–related signatures, including LDL and plasma lipoprotein clearance pathways (Figure 3A), showed a significant negative correlation (Spearman's R = −0.23; *P* = 0.04, and R = −0.23; *P* = 0.04, respectively; Figure 3A). Patients with a high proportion of activated DCs showed lower activity of the negative regulation of muscle hypertrophy (Figure 3A). To further explore whether activated DCs negatively correlated with pathways related to lipid clearance/muscle hypertrophy, we performed the above analyses in two datasets (GSE26081 and GSE26080). Notably, the content of activated DCs also negatively correlated with the signature of lipid clearance and muscle hypertrophy (Supplementary Figure 2A). The increase in Tfhs significantly correlated with the upregulated lipid biosynthetic process (Spearman's R = 0.3; *P* = 0.0073; Figure 3B). In contrast, this increase negatively correlated with the negative regulation of cellular extravasation (Spearman's R = −0.26; *P* = 0.022; Figure 3B). The score for negative regulation of glucocorticoid metabolic process negatively correlated with an increased proportion of Tfhs (Spearman's R = −0.24, *P* = 0.31; Figure 3B). These results showed a similar trend in these datasets (GSE26081 and GSE26080; Supplementary Figure 2B). Supplementary Figures 2C,D shows the correlation between the fractions of the two immune cell types and the activity of abnormal pathways. Overall, these results further indicated that CAD was associated with increased activation of DCs and Tfhs, the downregulated activity of lipid clearance and glucocorticoid metabolism, and the upregulated activity of cellular extravasation (Figure 3C). Notably, the proportion of Tregs and the score of these pathways, including metabolism of angiotensinogen to angiotensins, lipopolysaccharide, and smooth muscle cell migration, exhibited negative Spearman's correlations (R = −0.27, *P* = 0.017; R = −0.23, *P* = 0.036; and R = −0.25, *P* = 0.024, respectively; Figure 4A). We observed that the increased resting CD4+ T cells negatively correlated with the activity of some pathways related to ossification in bone remodeling and glutathione synthesis (R = −0.32; *P* = 0.0033; R = −0.34; *P* = 0.036; R = −0.25; *P* = 0.024; Figure 4B). There was a negative correlation between the fraction of γδ T cells and inflammation-related pathways, such as IL-23 signaling (R = −0.25; *P* = 0.025; Figure 4C) and NOTCH2 signaling (R = −0.25; *P* = 0.027; Figure 4C). Spearman correlation analysis showed similar results between these immune cell types (Tregs, resting CD4+ T cells and γδ T cells) and abnormal signaling signatures (Figure 4D and Supplementary Figures 4A–C). These results demonstrated that differentially abundant immune cells that are associated with abnormal signaling signatures may play an important role in the pathogenesis of CAD.

### Identification of Differential GEPs and Key Pathways in CAD

**Figure 5:**
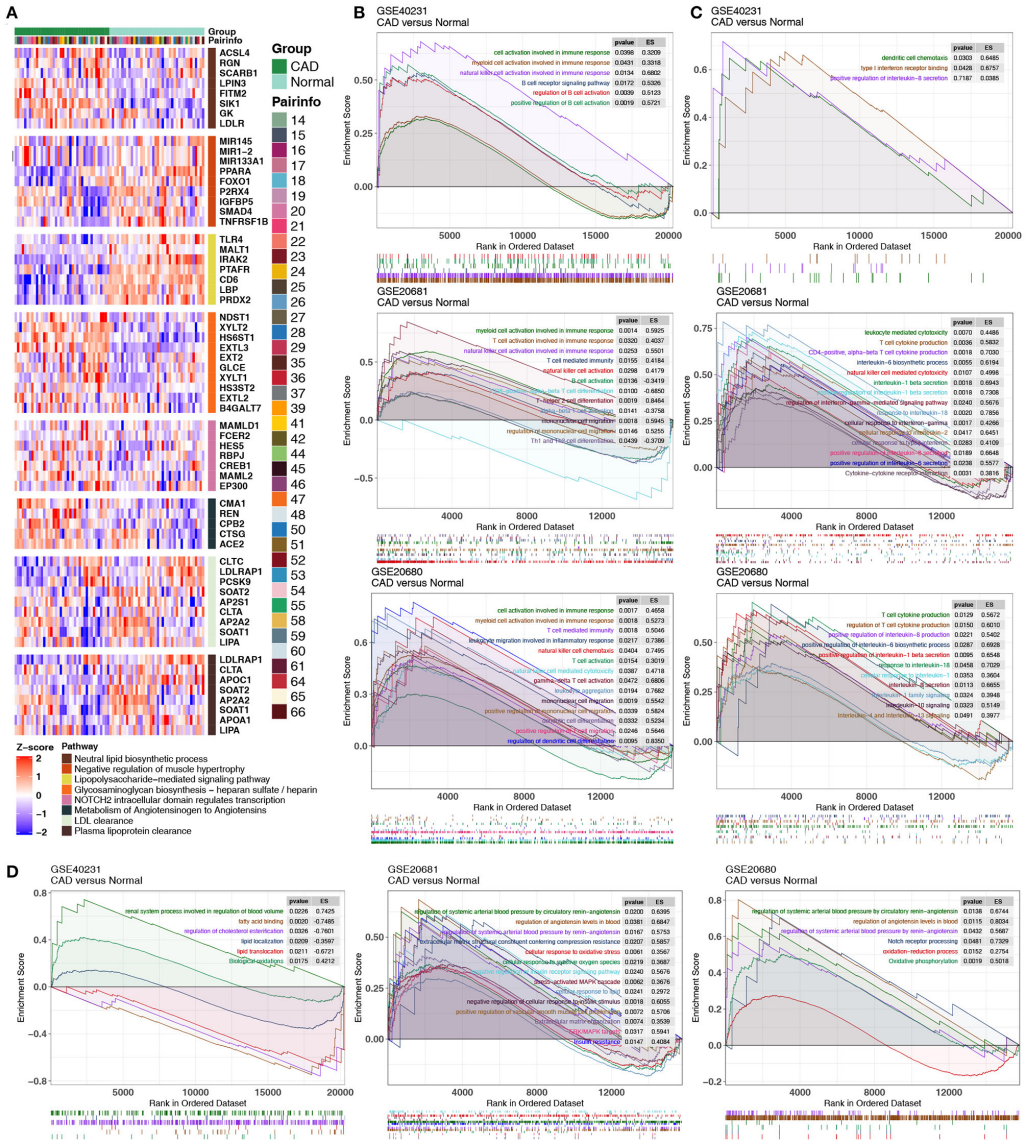
**(A)** Heatmap of core genes in significantly enriched pathways between the CAD cases vs. the healthy controls in GSE40231. **(B–D)** GSEA of hallmark gene sets in the CAD cases and the healthy controls downloaded from MSigDB (GSE40231, GSE20681, and GSE20680). CAD cases were associated with activated immune cell- **(B)**, cytokine- **(C)**, and inflammation-related pathways **(D)**. All transcripts were ranked as the log_2_ (fold-change) between the CAD cases and the healthy controls. Each run was performed with 1,000 permutations. CAD, coronary artery disease; MsigDB, Molecular signatures database.

We extracted the GEPs of hub genes in key pathways (related to Figures 3, 4) and compared these GEPs between the CAD and control groups in GSE40231. Genes related to lipid biosynthesis were significantly upregulated in the CAD group compared to the control group, and genes associated with lipid clearance signaling were significantly downregulated in the CAD group compared to the control group (Figure 5A). GSEA in three independent datasets indicated that immune cell-related signatures, such as natural killer cells (NKs), B cells, myeloid leukocytes, neutrophils, Th2, mononuclear cells, and CD8+ T cells were significantly enriched in the CAD group compared to the control group in all datasets (Figure 5B). Similarly, cytokine-related pathways, such as IL-1, IL-4, IL-6, IL-8, IL-13, IL-18, and IFN-γ signaling were significantly enriched in the CAD samples compared to the control group (Figure 5C). Endothelial cell morphogenesis, regulation of glomerular filtration, ROS metabolic process, insulin resistance, positive regulation of vascular smooth muscle cell proliferation, ERK/MAPK targets, and regulation of angiotensin levels in blood were significantly upregulated in the CAD group (Figure 5D).

## Discussion

**Figure 6:**
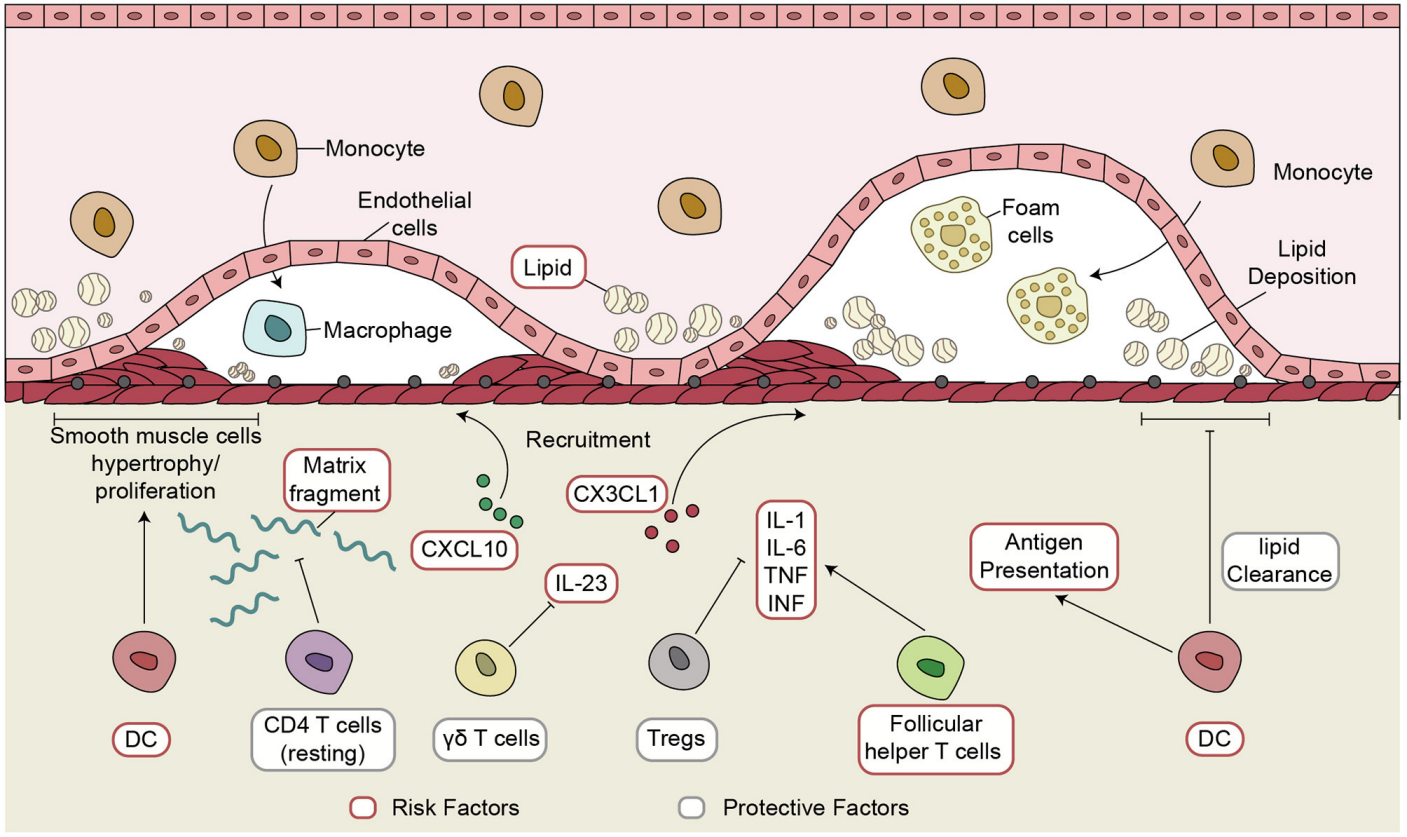
The possible inflammatory immune microenvironment in CAD. CAD, coronary artery disease.

We analyzed gene expression data of CAD patients and healthy controls from three independent datasets—a discovery dataset (GSE40231) and two validation datasets (GSE20681 and GSE60280)—to examine the immune microenvironment of coronary artery disease (CAD). CIBERSORT analysis revealed significant differences in five immune cells: activated DCs, Tfhs, resting CD4+ T cells, Tregs, and γδ T cells. Cell markers of these immune cells and immune-related functional genes were used for downstream analyses. Immune transcriptome analysis indicated that higher expression levels of inflammatory markers (CYT, antigen presentation, chemokines, and cytokines) were detected in the CAD group than in the controls. The ssGSEA algorithm based on the GSVA R package was used to calculate the activity of pathways that may be associated with the pathogenesis of CAD. The proportion of activated DCs and the lipid clearance signaling score showed significant negative correlations. We also observed a positive correlation between the fraction of Tfhs and the lipid biosynthetic signature. Spearman's correlation analysis revealed that the increased proportion of Tregs correlated with the downregulated activity of some pathways, such as metabolism of angiotensinogen to angiotensins, lipopolysaccharide, and smooth muscle cell migration. Notably, resting CD4+ T cells and the activity of some pathways related to ossification in bone remodeling and glutathione synthesis showed a negative correlation. The γδ T cells negatively correlated with IL-23 signaling activity. GSEA revealed a close association between the inflammatory immune microenvironment, including activated immune cells, chemokines and cytokines, and CAD. Therefore, we identified the immune microenvironment associated with inflammatory signaling based on the expression profiles of CAD cases. We summarized the possible mechanisms underlying the immune microenvironment in CAD (Figure 6).

DCs are the most powerful known antigen presenting cells and are a key link between innate immunity and acquired immunity. DCs play an important role in the occurrence and development of atherosclerosis (33). The release of proinflammatory cytokines, such as CCL5, CX3CL1, and CXCL10 promotes the migration of DCs (34–36). Mature and activated DCs accumulate locally with the progression of Aps (37). Liu et al. reported that the decreased expression of CX3CR1 and CX3CL1 and the downregulation of DCs narrowed plaques in CX3CR1^−/−^ApoE^−/−^ mouse models compared to healthy controls (38). The migration of DCs may also be impaired in hyperlipidemia (39), but the ability to stimulate these is not weakened (40), which may lead to a local accumulation of DCs and ultimately increase the inflammatory response, DC maturation, T cell activation, cytokine secretion (IL-1, IL-6, IL-8, INF-γ, and TNF), and TNF-α participation in the formation of atherosclerosis (41). Several recent trials focused on pro-inflammatory mediators in patients with CAD. The CANTOS (Canakinumab Anti-Inflammatory Thrombosis Outcome Study) trial was the most noteworthy of the studies and targeted IL-1β (42). The CANTOS trial provided favorable evidence that inhibition of the IL-1β/IL-6 signaling cascade led to a significant reduction in cardiovascular risk, independent of a lipid-lowering effect, but with an increased risk of serious infection (42). Experimental data in hyperlipidemic mice also revealed that inhibition of IL-6 or its receptor achieved atheroprotective effects (17). DCs may play an important role in the occurrence and development of atherosclerosis *via* lipid accumulation. Paulson et al. investigated the formation of early APs in Ldlr^−/−^ mice using immunofluorescence (43). After a high cholesterol diet, lipid accumulation in the blood vessels of CD11c+ DCs was induced. These DCs exhibited foam cell morphology and may participate in the early formation of plaques. The antigen presentation function of DCs plays an important role in the occurrence and development of atherosclerosis (44). MHC-I and MHC-II are known antigen-presenting molecules of DCs (44). DCs present antigens to T cells *via* MHC-I and MHC-II, which leads to the activation and proliferation of T cells (45). A previous study found that atherosclerosis was significantly decreased in Ldlr^−/−^CD74^−/−^ mice, and the activated T cells were also decreased. This finding may be related to the decrease in the presenting function of the DCs (46). DCs mainly take up and present specific atherosclerotic antigens in atherosclerosis. Arterial DCs activate DCs *via* ox-LDL uptake and increase the presentation of lipid and polypeptide antigens to T cells, thereby participating in the development of atherosclerosis (44). DCs regulate the process of atherosclerosis by controlling the activation of T cells (47). Koltsova et al. observed that DCs in ApoE^−/−^CD11c–YFP+ mice interacted with CD4+ T cells and resulted in T cell activation, proliferation, and the secretion of TNF-α and IFN-γ, which accelerated the process of atherosclerosis (47). Foam cells are one of the hallmarks of atherosclerotic plaques and develop when smooth muscle cells within the arterial wall take up ox-LDL *via* scavenger receptors (48). Consistent with previous findings (47), the results of the present study also suggest that activated DCs are associated with downregulated lipid clearance, increased antigen presentation, activation of cytotoxic responses, recruitment, and migration. Therefore, a critical protection mechanism toward avoiding immune cell infiltration into the vessel wall stems from wall-embedded DCs (49).

Tregs are a negative immune regulator that play an important anti-atherosclerotic role *via* inhibition of the autoimmune response and the maintenance of homeostasis of the body's immune response (50–52). Wigren et al. reported that individuals with low levels of Tregs were at increased risk of a first coronary event (53).

Hermansson et al. showed that an increase in Tregs was associated with a significant decrease in AP size (~70%), and a decrease in CD4+ T cell infiltration and systemic inflammation was observed (54). A previous study investigating the distribution of Tregs in human atherosclerotic lesions found that low numbers of Tregs were present during all developmental stages (55). Our data were consistent with the observation of the deficiency of Tregs in the experimental atherosclerosis model (56–58) and suggest decreased CD4+CD25+ Treg participation in plaque destabilization.

Tfhs cells promote B cell proliferation, differentiation, class conversion, and antibody production and the occurrence and development of atherosclerosis. Taghaviemoghadam et al. found decreased Tfhs and plasma cells in the spleen and aortic arch of STAT4^−/−^Ldlr^−/−^ mice compared to Ldlr^−/−^ mice, but CD8+ Tregs were increased, and the size of the APs decreased (59). A recent study showed that the expression of Foxp3 in Tregs and the immunosuppressive function were lost during the process of atherosclerosis, which resulted in the transformation of some Tregs into Tfhs. Tfhs promote atherosclerosis, and Tfh depletion alleviates atherosclerosis, and ApoAI prevents Tregs from transforming into Tfhs, which affects the development of atherosclerosis (60). Tfhs in the peripheral blood of CAD patients are significantly higher than healthy populations and have a stronger proinflammatory function. An *in vitro* co-culture revealed a significant increase in the expression of INF-γ, IL-17, and IL-21 in coronary heart disease patients, and an increase in the expression level of B cell inflammatory genes (61). Our results suggest that CAD patients have a decreased proportion of γδ T cells, which negatively correlated with IL-23 signaling. A previous study showed that the expression level of IL-23 in the peripheral blood of CAD patients was significantly higher than that in healthy people. The high expression level of IL-23 was associated with a higher mortality of CAD patients (5). These results provide potential targets of inflammation to improve outcomes in atherosclerotic cardiovascular disease.

Although the findings in the present study systematically summarized the immune microenvironment of CAD cases based on their expression profiles using multiple bioinformatics methods, the results should be validated in prospective studies with larger populations. The present study also had some limitations. First, cell experiments and animal experiments are lacking. Second, this study was based solely on gene expression arrays (bulk transcriptome). All datasets in this study lacked clinical data, which may have potential confounders. This report lacks other omics analyses to further validate our results, and the traditional bulk array may obscure the heterogeneity of various immune cells in CAD. Third, there were limited tissue data for CAD, and the present study used two blood dataset and one tissue dataset of CAD to further validate the immune microenvironment. Fourth, the related clinical characteristics are lacking, a subgroup analysis will be conducted in the future using prospective cohorts.

## Conclusions

The present study revealed that CAD patients may have an inflammatory immune microenvironment. The study also provides a timely update on inflammatory components in the immune microenvironment of patients with CAD.

## Data Availability Statement

The original contributions presented in the study are included in the article/Supplementary Material, further inquiries can be directed to the corresponding author/s.

## Author Contributions

Conceptualization, formal analysis, supervision, and visualization: HKH and RSD. Software: HKH, RSD, and PC. Writing–original draft: HKH, RSD, PC, JCZ, YC, and GL. Writing–review & editing: HKH, RSD, YC, and GL. All authors contributed to the article and approved the submitted version.

## Conflict of Interest

The authors declare that the research was conducted in the absence of any commercial or financial relationships that could be construed as a potential conflict of interest.

## Supplementary Material

Supplementary Figure 1 The boxplot of gene expression profiles in each of the three datasets before and after normalization.

Supplementary Figure 2 The correlation between the proportions of activated DCs and the score of ssGSEA in the two validation GEO datasets (GSE20681 and GSE20680). The correlation between the proportions of Tfhs and the score of ssGSEA in each validation GEO dataset (GSE20681 and GSE20680). The correlation heatmap between the proportions of activated DCs and the score of ssGSEA in the two validation GEO datasets (GSE20681 and GSE20680). The correlation heatmap between the proportions of Tfhs and the score of ssGSEA in each validation GEO dataset (GSE20681 and GSE20680). DCs, dendritic cells; ssGSEA, enrichment analysis; GEO, Gene Expression Omnibus database; Tfhs, T follicular helper cells.

Supplementary Figure 3 The correlation between the proportions of Tregs and the score of ssGSEA in the two validation GEO datasets (GSE20681 and GSE20680). The correlation between the proportions of resting CD4+ T cells and the score of ssGSEA in the two validation GEO datasets (GSE20681 and GSE20680). The correlation between the proportions of γδ T cells and the score of ssGSEA in GSE20681. Tregs, regulatory T cells; ssGSEA, enrichment analysis; GEO, Gene Expression Omnibus database; γδ T cells, gamma delta T cells.

Supplementary Figure 4 The correlation heatmap between the proportions of Tregs and the score of ssGSEA in the two validation GEO datasets (GSE20681 and GSE20680). The correlation heatmap between the proportions of resting CD4+ T cells and the score of ssGSEA in the two validation GEO datasets (GSE20681 and GSE20680). The correlation heatmap between the proportions of γδ T cells and the score of ssGSEA in GSE20681. ssGSEA, enrichment analysis; GEO, Gene Expression Omnibus database; γδ T cells, gamma delta T cells.

## Abbreviations

CAD: coronary artery disease
DCs: dendritic cells
Tfhs: T follicular helper cells
Tregs: regulatory T cells
AS: Atherosclerosis
APs: atherosclerotic plaques
TNF: tumor necrosis factor
ROS: reactive oxygen species
GEO: Gene Expression Omnibus database
CYT: cytolytic activity
ssGSEA: single-sample gene set enrichment analysis
GSEA: gene set enrichment analysis
GEP: gene expression profile
γδ T cells: gamma delta T cells
NKs: natural killer cells
LDL: low density lipoprotein.
