# Supplementary figures and images for: Comprehensive Analysis of the Immune Infiltrates and Aberrant Pathways Activation in Atherosclerotic Plaque
Source: Front Cardiovasc Med. 2021 Feb 5;7:602345. doi: 10.3389/fcvm.2020.602345 (PMC7892440; doi:10.3389/fcvm.2020.602345)

# GSE20681

**A**

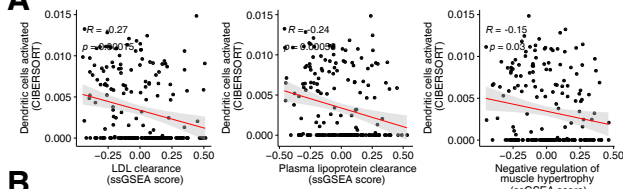

**B**

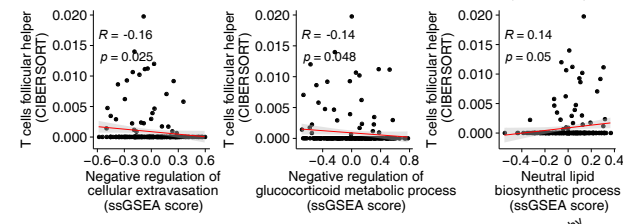

**C**

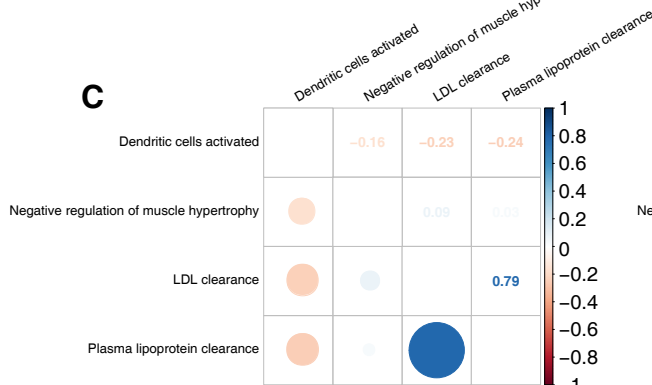

**D**

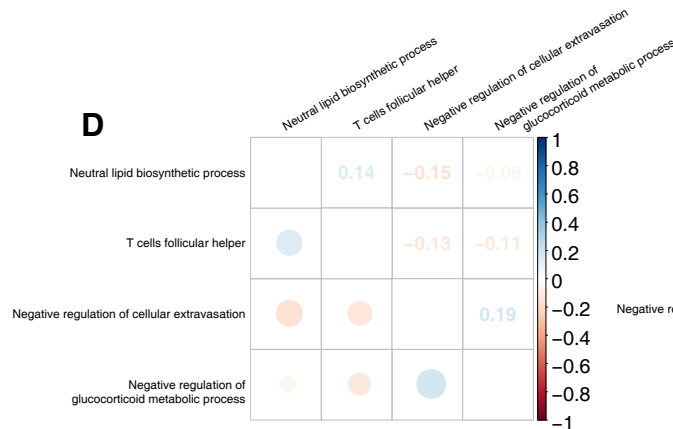

# GSE20680

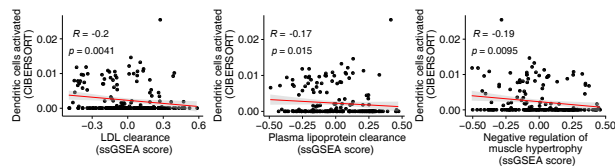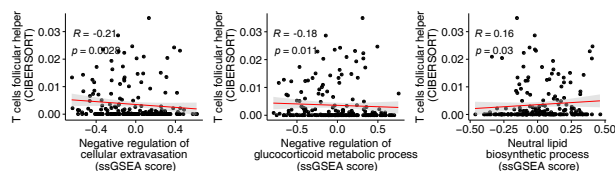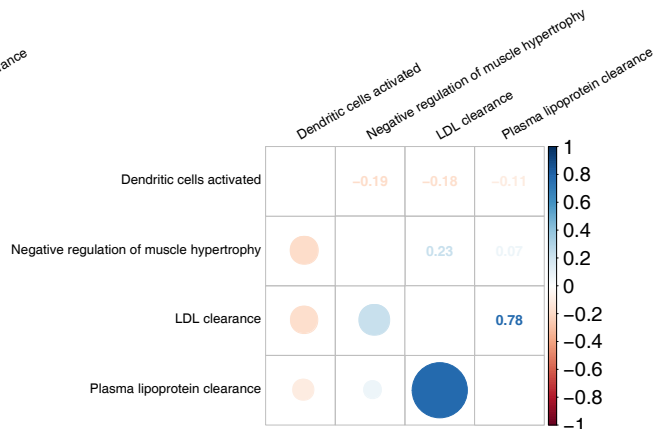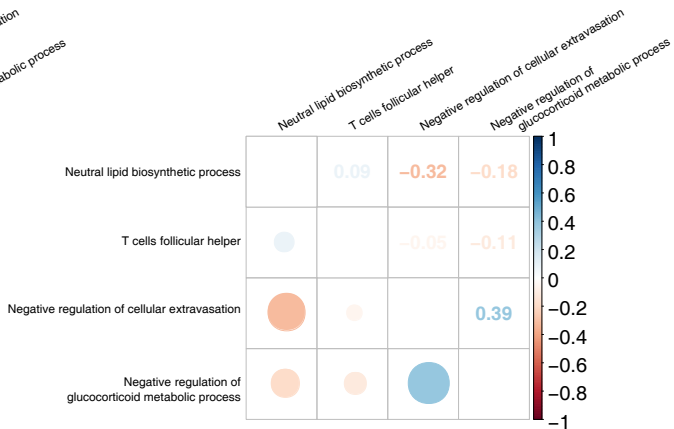

Supplement: Supplementary Figure 2 — The correlation between the proportions of activated DCs and the score of ssGSEA in the two validation GEO datasets (GSE20681 and GSE20680). The correlation between the proportions of Tfhs and the score of ssGSEA in each validation GEO dataset (GSE20681 and GSE20680). The correlation heatmap between the proportions of activated DCs and the score of ssGSEA in the two validation GEO datasets (GSE20681 and GSE20680). The correlation heatmap between the proportions of Tfhs and the score of ssGSEA in each validation GEO dataset (GSE20681 and GSE20680). DCs, dendritic cells; ssGSEA, enrichment analysis; GEO, Gene Expression Omnibus database; Tfhs, T follicular helper cells. [file Data_Sheet_2.PDF]

# GSE20681

**A**

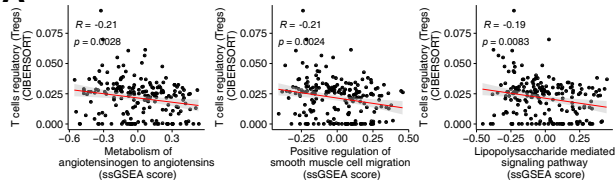

**B**

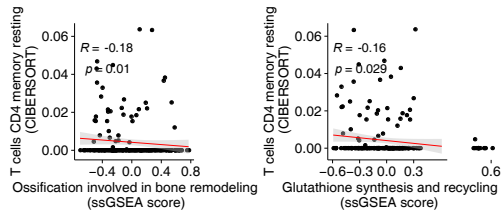

**C**

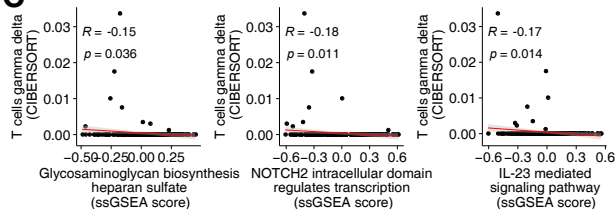

# GSE20680

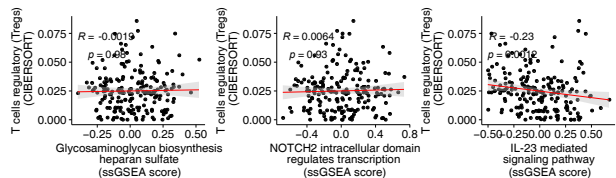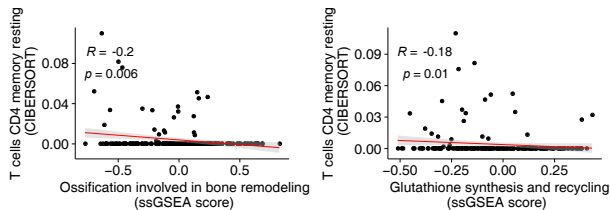

Supplement: Supplementary Figure 3 — The correlation between the proportions of Tregs and the score of ssGSEA in the two validation GEO datasets (GSE20681 and GSE20680). The correlation between the proportions of resting CD4+ T cells and the score of ssGSEA in the two validation GEO datasets (GSE20681 and GSE20680). The correlation between the proportions of γδ T cells and the score of ssGSEA in GSE20681. Tregs, regulatory T cells; ssGSEA, enrichment analysis; GEO, Gene Expression Omnibus database; γδ T cells, gamma delta T cells. [file Data_Sheet_3.PDF]

A

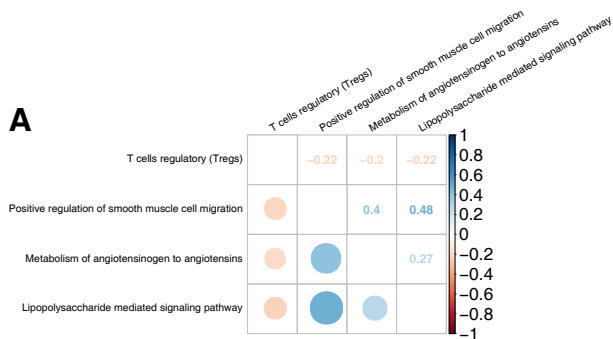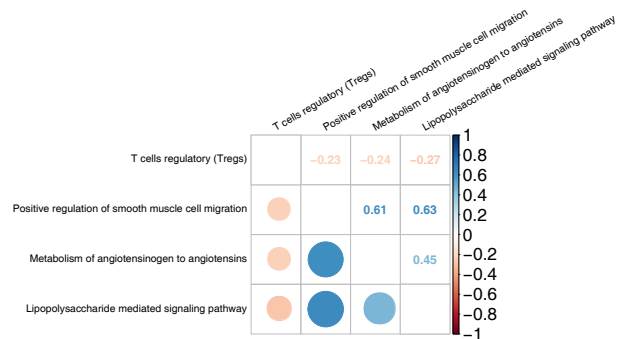

B

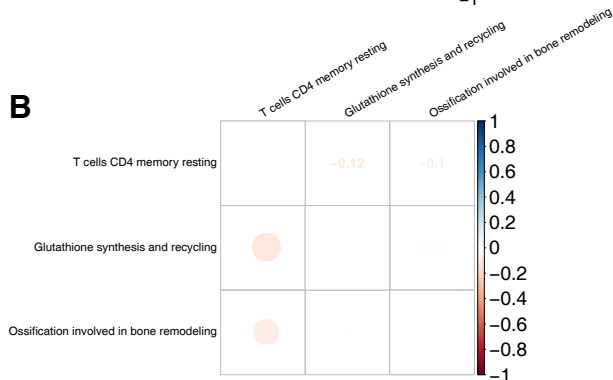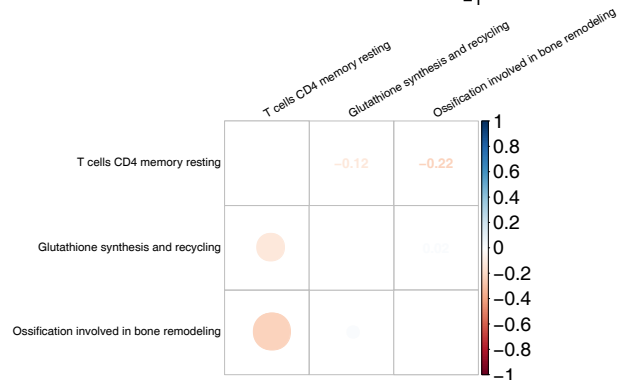

C

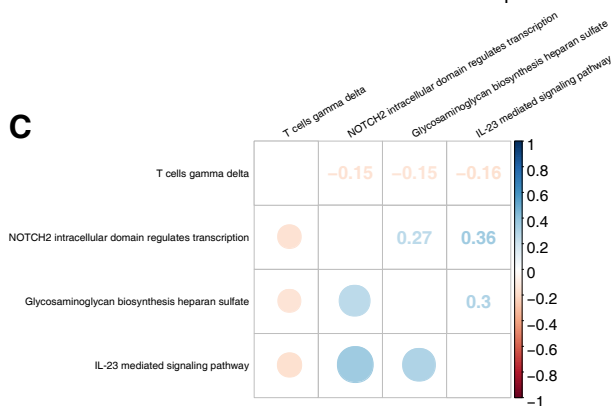

Supplement: Supplementary Figure 4 — The correlation heatmap between the proportions of Tregs and the score of ssGSEA in the two validation GEO datasets (GSE20681 and GSE20680). The correlation heatmap between the proportions of resting CD4+ T cells and the score of ssGSEA in the two validation GEO datasets (GSE20681 and GSE20680). The correlation heatmap between the proportions of γδ T cells and the score of ssGSEA in GSE20681. ssGSEA, enrichment analysis; GEO, Gene Expression Omnibus database; γδ T cells, gamma delta T cells. [file Data_Sheet_4.PDF]
