# Supplementary material for: Comprehensive Analysis of the Immune Infiltrates and Aberrant Pathways Activation in Atherosclerotic Plaque
Source: Front Cardiovasc Med. 2021 Feb 5;7:602345. doi: 10.3389/fcvm.2020.602345 (PMC7892440; doi:10.3389/fcvm.2020.602345)
Supplement: Supplementary Figure 1 — The boxplot of gene expression profiles in each of the three datasets before and after normalization. [file Data_Sheet_1.PDF]

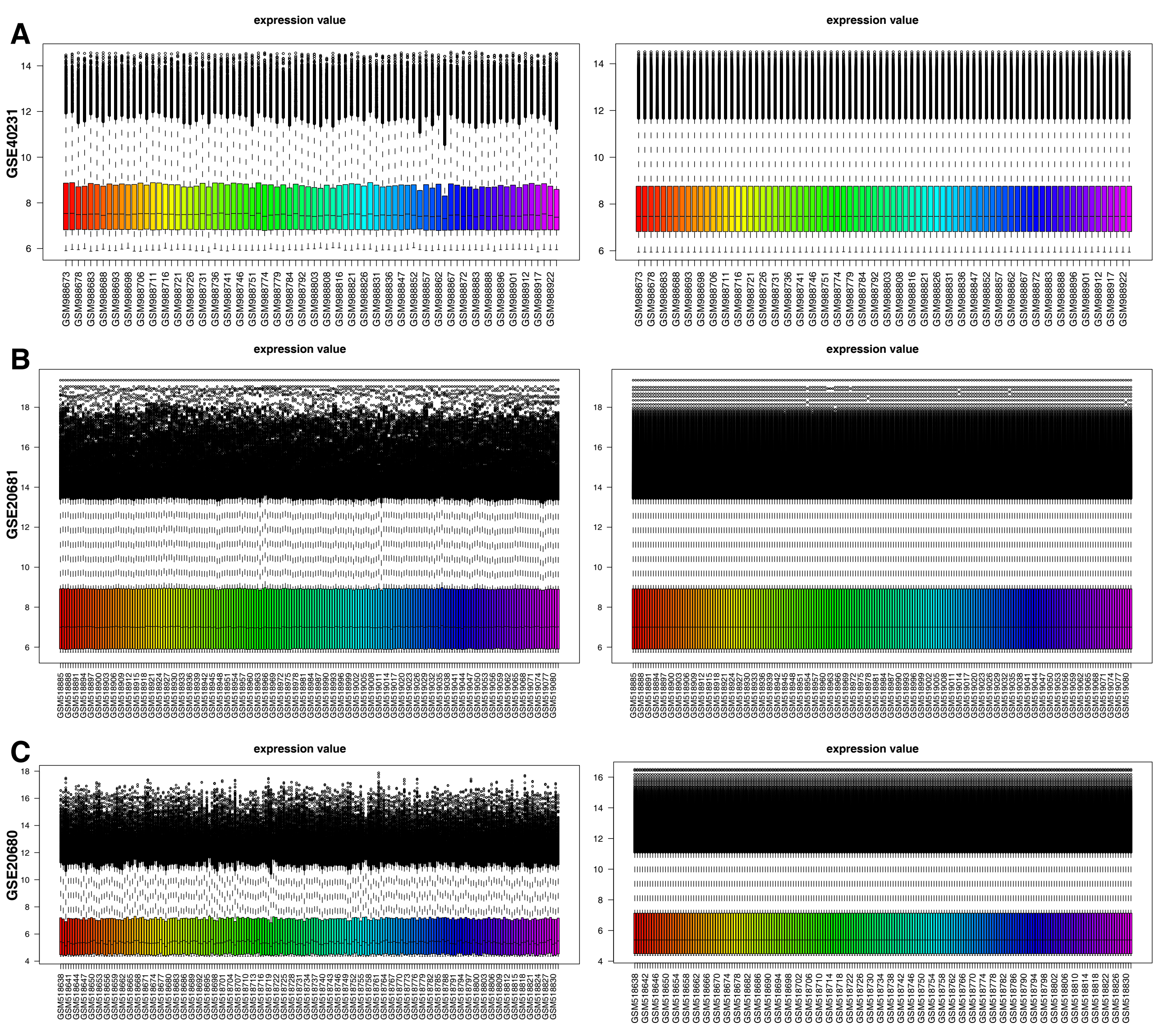
A
B
C
expression value
GSE40231
GSE20681
GSE20680
GSM988673 GSM988678 GSM988683 GSM988688 GSM988693 GSM988698 GSM988706 GSM988711 GSM988716 GSM988721 GSM988726 GSM988731 GSM988736 GSM988741 GSM988746 GSM988751 GSM988774 GSM988779 GSM988784 GSM988792 GSM988803 GSM988808 GSM988816 GSM988821 GSM988826 GSM988831 GSM988836 GSM988847 GSM988852 GSM988857 GSM988862 GSM988867 GSM988872 GSM988883 GSM988888 GSM988896 GSM988901 GSM988912 GSM988917 GSM988922
